# Differential eco-physiological performance to declining groundwater depth in Central Asian C_3_ and C_4_ shrubs in the Gurbantunggut Desert

**DOI:** 10.3389/fpls.2023.1244555

**Published:** 2024-01-18

**Authors:** Bahejiayinaer Tiemuerbieke, Jian-Ying Ma, Wei Sun

**Affiliations:** ^1^ Xinjiang Key Laboratory of Oasis Ecology, College of Geography and Remote Sensing Sciences, Xinjiang University, Urumqi, China; ^2^ Key Laboratory of Geographical Processes and Ecological Security in Changbai Mountains, Ministry of Education, School of Geographical Sciences, Northeast Normal University, Changchun, China; ^3^ Key Laboratory of Vegetation Ecology, Ministry of Education, Institute of Grassland Science, Northeast Normal University, Changchun, China

**Keywords:** C_3_ and C_4_ photosynthesis, desert shrubs, eco-physiological acclimation, water-carbon balance, drought stress

## Abstract

Resources in water-limited ecosystems are highly variable and unpredictable, and the maintenance of functional diversity among coexisting species is a crucial ecological strategy through which plants mitigate environmental stress. The comparison of differential eco-physiological responses among co-occurring plants in harsh environments could help provide deep insights into the coexistence mechanisms of competing species. Two coexisting desert shrubs with different photosynthetic pathways (*Haloxylon ammodendron* and *Tamarix ramosissima*) were selected in the Gurbantunggut Desert located in northwest China. This study detected variations in the water sources, photosynthetic parameters, stem water status, and non-structural carbohydrates of the two shrubs at three sites with different groundwater table depths during the growing seasons of 2015 and 2016 to identify distinct eco-physiological performances in coexisting plants with different functional types under fluctuating water conditions. The water sources of *H. ammodendron* shifted from soil water to groundwater, while *T. ramosissima* extracted water mainly from deep soil layers at both sites. Significant reductions in carbon assimilation and stomatal conductance in *H. ammodendron* with deeper groundwater table depth were detected during most drought periods, but no significant decreases in transpiration rate were detected with declining groundwater table depth. For *T. ramosissima*, all of these gas exchange parameters decreased with the progression of summer drought, and their relative reduction rates were larger compared with those of *H. ammodendron*. The stem water status of *H. ammodendron* deteriorated, and the relative reduction rates of water potential increased with deeper groundwater, whereas those of *T. ramosissima* did not differ with greater groundwater depth. These findings indicated that prolonged drought would intensify the impact of declining groundwater depth on the eco-physiology of both shrubs, but the extent to which the shrubs would respond differed. The two shrubs were segregated along the water–carbon balance continuum: the C_3_ shrub *T. ramosissima* maximized its carbon fixation at an enormous cost of water, while greater carbon fixation was achieved with far greater water economy for *H. ammodendron*. These results demonstrated that the two shrubs prioritized carbon gain and water loss differently when faced with limited water sources. These mechanisms might mitigate competitive stress and enable their coexistence.

## 1 Introduction

Water source availability is the most determining factor in plant survival and ecosystem functioning in arid ecosystems. As a main water input, precipitation is typically insufficient, unpredictable, and inconsistent in arid environments (Schwinning and Ehleringer, 2001), causing the soil moisture to fluctuate with precipitation. As a result, desert plants heavily rely on groundwater as one of the few reliable sources of water (Wu et al., 2019). However, the groundwater resources of arid regions face critical threats such as overexploitation and contamination due to climate change and anthropogenic activities (Panneerselvam et al., 2022). General circulation models have predicted the unprecedented duration, intensity, and seasonality of future droughts, which may alter the precipitation patterns of arid areas (Huang et al., 2016; Bhusal et al., 2020). This trend can significantly affect water resource accessibility, which may alter the composition and function of arid ecosystems (Hoover et al., 2014; Grossiord et al., 2017). Obtaining a deeper understanding of desert ecosystem responses to varying water conditions will be essential to ecosystem protection and the assessment of ecosystem adaptation strategies under future climate change scenarios.

In drylands, diversity in functional types is essential for the coexistence of plants, through which plants can use limited resources efficiently, as well as differently in space and time to cope with periodic and chronic drought events (Chesson et al., 2004; Araya et al., 2011; Pardos et al., 2021). Differential mechanisms adopted by coexisting plants have been examined extensively to explain how competing species in water-limited environments achieve stable coexistence (Angert et al., 2009; Adler et al., 2013; Bermúdez and Retuerto, 2014). Resource acquisition traits (root functioning) are among the most important mechanisms that can help desert plants partition their water sources spatially and temporally to alleviate competition stress for limited water resources (Filella and Penuelas, 2003; Eggemeyer et al., 2008; Ellsworth and Sternberg, 2015). Plant coexistence mechanisms are also closely related to the resource use strategies (leaf and shoot function and traits) of different plants (Moreno-Gutierrez et al., 2012). The species-specific adaptations of different plants to a certain environment are modulated by their own evolutionary trajectories; water-use strategies and trade-offs are, consequently, inherent to different species (Filella and Penuelas, 2003). Therefore, the knowledge of variations in eco-physiological responses and the water–carbon trade-offs of coexisting plants help to reveal the differential eco-physiological acclimation and coexistence mechanisms of competing species.

C_3_ and C_4_ plants are two differential functional types that respond differently to environmental stress due to contrasting water–carbon balance. C_4_ photosynthesis refers to various modifications in anatomy, biochemistry, and physiology that strategically concentrate CO_2_ in the bundle sheath, which leads to the saturation of Rubisco at ambient CO_2_ concentrations (Sage and Pearcy, 1987; Taylor et al., 2011; Osborne and Sack, 2012; Taylor et al., 2014). C_4_ plants are able to increase their maximum net photosynthesis rates with less stomatal conductance compared to C_3_ species by almost eliminating photorespiration. As a result, they can reduce transpiration and conserve water, which is particularly beneficial in hot conditions with high evaporative demand (Pearcy and Ehleringer, 1984; Osborne and Sack, 2012; Taylor et al., 2014). Therefore, C_4_ photosynthetic traits and resultant increased water-use efficiency are likely to provide C_4_ plants with selective and competitive advantages over C_3_ plants in hot and arid climates (Ripley et al., 2007; Ripley et al., 2010). These aforementioned observations bring up necessary inquiries regarding the benefits and costs of C_4_ photosynthesis in water-limited ecosystems.

*Tamarix ramosissima* and *Haloxylon ammodendron* are the dominant desert shrubs with C_3_ and C_4_ photosynthetic pathways, respectively, that are distributed in the Gurbantunggut Desert of Central Asia. These two desert shrubs play critical roles in maintaining desert ecosystem structure and functioning, biodiversity conservation, and soil protection. Despite the importance of the shrubs, there remains a paucity of extensive evidence on the responses of C_3_ and C_4_ desert shrubs to the changing groundwater depth. Previous works have mainly focused on observations of the precipitation responses and water resource use of these C_3_ and C_4_ desert shrubs, thus limiting the understanding of these shrubs and hindering the ability of researchers to predict future impacts on the Central Asian Desert ecosystem. With differential photosynthetic pathways, the two shrubs might develop contrasting forms of eco-physiological acclimation, as well as different response patterns under changing water conditions. This study investigated the eco-physiological performances of the two shrubs under declining groundwater depths to detect their divergent survival mechanisms in an unfavorable environment. This study aimed to address the following questions: 1) to what degree do the two shrubs rely on groundwater? 2) To what extent does the varying groundwater depth affect the eco-physiological performance of the two desert shrubs? 3) Does the C_4_ pathway have any advantages under drought stress?

## 2 Materials and methods

### 2.1 Study sites and experimental design

The study area was on the southern border of the Gurbantunggut Desert located in the Central Asian continental desert, which was near the Fukang Station of Desert Ecology, Chinese Academy of Sciences (44°22′N, 87°55′E). The research area has a typical temperate continental arid climate, with a hot, dry summer lasting from June to the end of August, and a cold, snow-covered winter lasting from December to March. The mean annual temperature is 6.6°C (1997–2016), the annual potential evaporation is approximately 1,000 mm, and the annual precipitation ranges from 70 to 180 mm (Fan et al., 2014; Xu et al., 2016). The C_3_ and C_4_ shrubs are the dominant vegetation types distributed across the area from the fringe of the alluvial plain to the interior of the desert.

**Figure 1:**
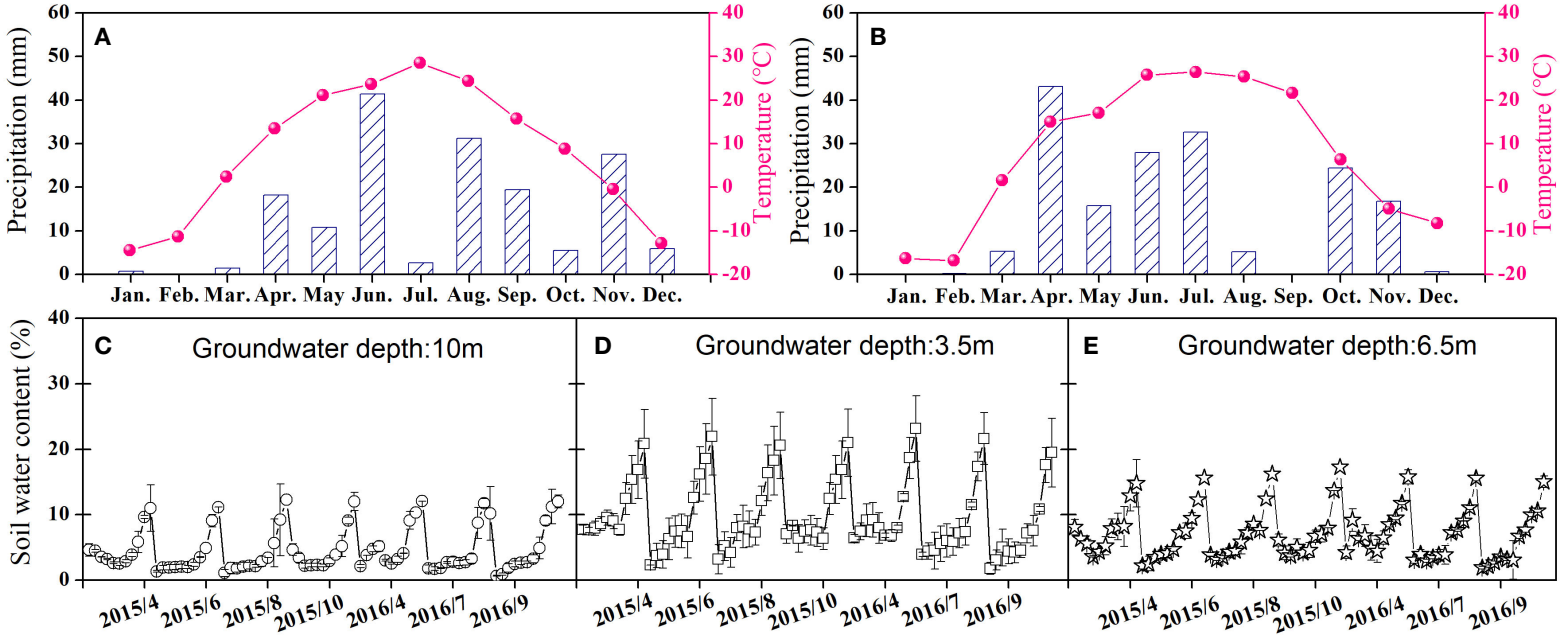
Variations in monthly total precipitation (mm) and monthly average temperature (°C) of 2015 **(A)** and 2016 **(B)** in the southern edge of Gurbantunggut Desert and gravimetric soil water content of the three different study sites (**C** represents the C_4_ site; **D** represents C_3_/C_4_ site; **E** represents C_3_ site) during the growing seasons of 2015 and 2016.

Three shrub communities with distinct vegetation structures were selected along the groundwater gradient from the southern border to the interior of the desert to identify the divergent eco-physiological performances of two different C_3_ and C_4_ shrubs to deteriorating soil water conditions. The C_4_ shrub community (C_4_ site hereafter) was chosen from the interior of the sandy desert, where the groundwater depth was approximately 10 m. The C_3_ and C_4_ mixed shrub community (C_3_/C_4_ site hereafter) and the C_3_ shrub community (C_3_ site hereafter) were chosen from the fringe of the alluvial plain, where the groundwater depths were 3.5 m and 6.5 m, respectively (**Figure 1**). The selected three sites had comparable climatic characteristics and weather patterns and were 5–8 km apart. The C_4_ shrub *H. ammodendron* (C. A. Mey.) Bunge was chosen in the C_4_ site, and the C_3_ shrub *T. ramosissima* Ledeb. Fl. Alt. was selected in the C_3_ site, and both shrubs were chosen from the C_3_/C_4_ site for comparison. The characteristics of these shrubs are presented in **Supplementary Table 1**. The study was conducted for two consecutive years, 2015 and 2016, with similar annual precipitation amounts (approximately 170 mm) and mean annual temperatures (**Figure 1**). The soil moisture differed with the groundwater depth in the selected three shrub communities, with the C_3_/C_4_ site having the highest soil moisture and the C_4_ site having the lowest soil moisture (**Figure 1**).

### 2.2 Sampling and water source analysis

Precipitation, soil, plant stems, and groundwater samples were collected to determine the plant water sources. Soil, stem, and groundwater samples were collected in April, June, August, and October 2015 and in April, July, and September 2016. Precipitation samples were collected after each rainfall (or snowfall) event using a collector installed in the ground of each site. The meteorological data were collected from the weather station located in the study area. Detailed information on the soil and stem sample collection method and soil water content measurement were described by Tiemuerbieke et al. (2018). Soil and xylem water were extracted using a cryogenic vacuum distillation system, and the oxygen and hydrogen isotope compositions of all the water samples were analyzed using a liquid water isotope analyzer (LWIA, DLT-100, Los Gatos Research Inc., Mountain View, CA, USA), with precision levels of 0.25‰ and 0.1‰ for δ^18^O and δ^2^H, respectively. The specific procedures were described by Tiemuerbieke et al. (2018) and Wu et al. (2014).

### 2.3 Leaf gas exchange measurements

A portable gas exchange system (Li-6400; Li-Cor, Lincoln, NE, USA) was used to measure the leaf gas exchange parameters of C_3_ and C_4_ shrubs monthly from June to September. This study detected the responses of leaf gas exchange to photosynthetically active radiation and intercellular CO_2_ concentration from 08:00 to 14:00 on clear sunny days. To measure the light response of plants, the light source leaf chamber (20 × 30 mm^2^) was used to measure the light response of three to four mature, fully extended sunlit leaves from each tree, and the light intensities were set at 0 μmol m^−2^ s^−1^, 20 μmol m^−2^ s^−1^, 50 μmol m^−2^ s^−1^, 100 μmol m^−2^ s^−1^, 150 μmol m^−2^ s^−1^, 200 μmol m^−2^ s^−1^, 400 μmol m^−2^ s^−1^, 600 μmol m^−2^ s^−1^, 800 μmol m^−2^ s^−1^, 1,200 μmol m^−2^ s^−1^, 1,600 μmol m^−2^ s^−1^, 1,800 μmol m^−2^ s^−1^, 2,000 μmol m^−2^ s^−1^, and 2,200 μmol m^−2^ s^−1^. The chamber temperature was adjusted to 30°C to keep the ambient air temperature and relative humidity, and the gas flow rate was set at 400 μmol/s with a 400 μmol/mol reference CO_2_ concentration. A modified rectangular hyperbola model (Ye and Yu, 2008) was used to derive the light saturation point (*LSP*) maximum net photosynthetic rate (*P_nmax_*):


(1) $$P_{n\max}=\alpha \frac{1-\beta I}{1+\gamma I}I-R_{d}$$

where *P_nmax_* represents the maximum net photosynthetic rate; *I* represents the PPFD_i_; *α* represents the initial slope of the photosynthetic light-response curve when PPFD_i_ approaches zero, namely, the apparent quantum efficiency; *β* and *γ* are coefficients that are independent of *I* and obtained by curve fitting (Ye & Yu, 2008); and Rd represents the dark respiration. The stomatal conductance (*g_s_*) and transpiration rate (*T_r_*) were measured at PPFD_i_ = 1,800 μmol m^−2^ s^−1^. Nine measurements were performed for each species, with three duplicates for each species at each site.

### 2.4 Stem water potential measurements

A pressure chamber (Model 3005, PMS Instrument Company, Albany, NY, USA) was used to test the stem water potential for three duplicates of shrubs at each site (a total of nine measurements were taken for each species) from June to September of 2015 and in June, August, and September of 2016. The specific procedures were described by Tiemuerbieke et al. (2018).

### 2.5 Non-structural carbohydrate analysis

The leaf sample collection for leaf non-structural carbohydrate (NSC) analysis was conducted simultaneously with gas exchange measurements. After being collected, the plant leaves were frozen immediately in liquid nitrogen to halt any enzymatic activity. Then, samples were dried in the laboratory at 65°C for 48 h before being ground to a fine powder to measure NSC concentrations, including leaf solute sugars and starch. This study used a modified microplate method (Zhao et al., 2010) to analyze foliar NSC, including foliar solute sucrose and starch content. To extract ethanol-soluble sucrose, the samples were heated in an 80°C water bath for 15 minutes. A mixture of 70 mg of ground tissue and 2 mL of 80% (v/v) ethanol was utilized for the extraction process. The starch concentration was determined by measuring the glucose in an aliquot of the supernatant following the hydrolysis of starch in the sample residue remaining after the EtOH extraction of soluble NSC fractions. The specific procedures were described by Zhao et al. (2010).

### 2.6 Data analysis

The inter- and intraspecific differences, seasonal (or monthly) differences in water potential, gas exchange parameters, and foliar non-structural carbohydrate content between the two plants were tested using univariate analysis based on a general linear model (GLM) and one-way analysis of variance (ANOVA), in which the species, season (or month), and sites were set as fixed effects. Prior to analysis, normality and homoscedasticity tests were conducted on all data, and transformations (logarithmic or square root) were applied as needed. For *post hoc* tests, Tukey’s honestly significant difference (HSD) was utilized. Pearson’s correlation coefficient was employed for correlation analysis. All data analyses were carried out using SPSS 19.0 (SPSS Inc., Chicago, IL, USA), while linear and non-linear regression analyses and graphing were performed using Origin 8.5 (Origin Lab Corp., Northampton, MA, USA).

The relative reduction ratios (*RRR*s) for some of the parameters were calculated to evaluate the impact of groundwater decline on the plant’s physiological performance:


(2) $$RRR=\frac{B-A}{B}\times 100\%$$

where *B* is the mean value of the parameters at the shallower groundwater depth site and *A* is the mean value of the parameters at the deeper groundwater depth site. Therefore, this method evaluates the relatively reduction ratio of given parameters at the site with deeper groundwater table depth compared to those of the site with shallower groundwater table depth.

## 3 Results

### 3.1 Variations in the water sources

**Figure 2:**
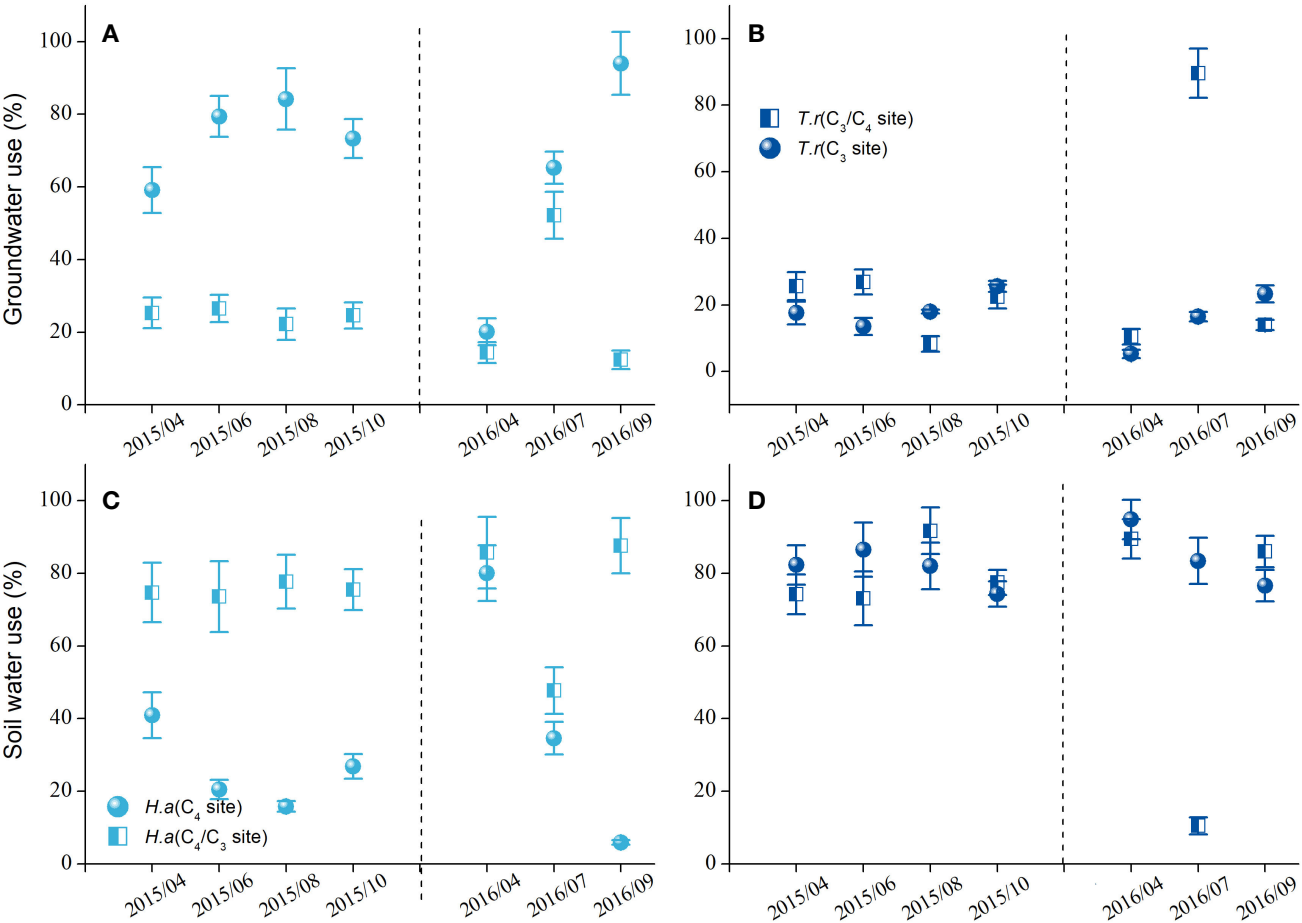
The percentage of groundwater **(A, B)** and soil water use **(C, D)** of the *Haloxylon ammodendron* and *Tamarix ramosissima* at the sites with different groundwater table depths during the growing seasons of 2015 and 2016.

The water sources of the *H. ammodendron* differed at the two sites with different groundwater table depths (**Figure 2**). The percentage of groundwater use was higher for the *H. ammodendron* at the C_4_ site, whereas the percentage of soil water was higher for the *H. ammodendron* at the C_3_/C_4_ site (**Figure 2**). However, the water sources of *T. ramosissima* did not differ between the two sites with different groundwater table depths, and the shrub mainly used soil water over groundwater at both sites (**Figure 2**).

### 3.2 Responses in gas exchange parameters

**Figure 3:**
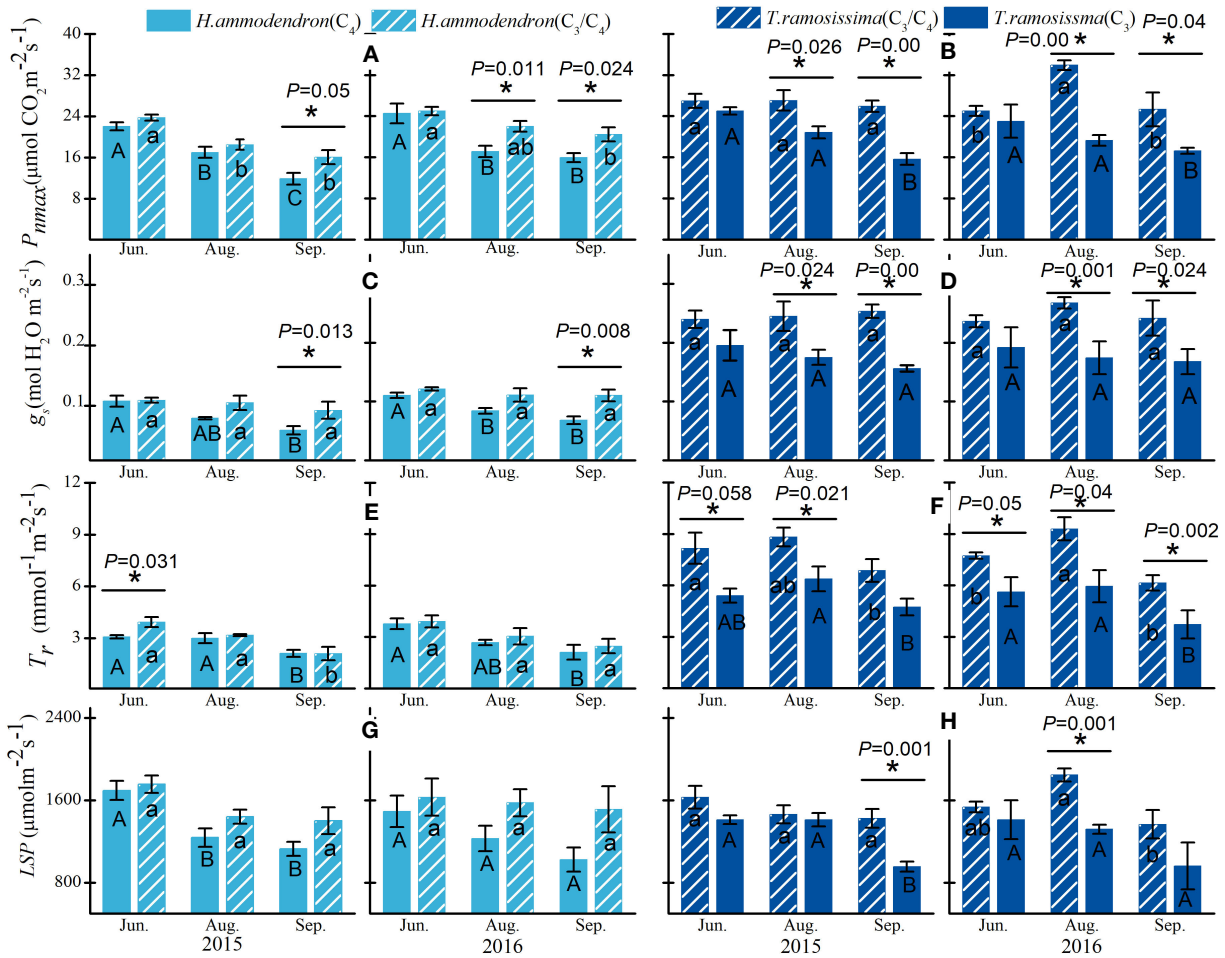
Variations in maximum net photosynthetic rate (*P_nmax_*) and stomata conductance (*g_s_*), transpiration rate (*T_r_*), and *LSP* of *Haloxylon ammodendron* **(A, C, E, G)** and *Tamarix ramosissima* **(B, D, F, H)** in different sites during the growing seasons of 2015 and 2016. The different uppercase and lowercase letters represent monthly significant differences for given shrubs at different sites. * represents significant intraspecific differences in different sites (*p*< 0.05). Data are presented as mean ± 1 standard error (n = 6).

Significant differences were detected in the gas exchange parameters between the shrubs at different sites for both *H. ammodendron* and *T. ramosissima* (**Figure 3**). The significant differences in the P*nmax* (Equation 1) and gs of *H. ammodendron* in the two sites varied with months, with the P*nmax* and g*s* of *H. ammodendron* in the C4 site reduced significantly during August and September compared with the *H. ammodendron* in the C3/C4 site (**Figure 3**). The *LSP* and *T_r_* of *H. ammodendron* at the C_4_ site exhibited a decreasing trend from June to September, but the differences were not statistically significant (**Figure 3**). For the *T. ramosissima*, decreases in the *P_nmax_*, *g_s_*, *T_r_*, and *LSP* of *T. ramosissima* in the drier site (C_3_ site) were detected during the entire study period of both years, and the reductions in *P_nmax_*, *g_s_*, and *LSP* went greater during the drier months of both years (**Figure 2**).

### 3.3 Variation in stem water potential

**Figure 4:**
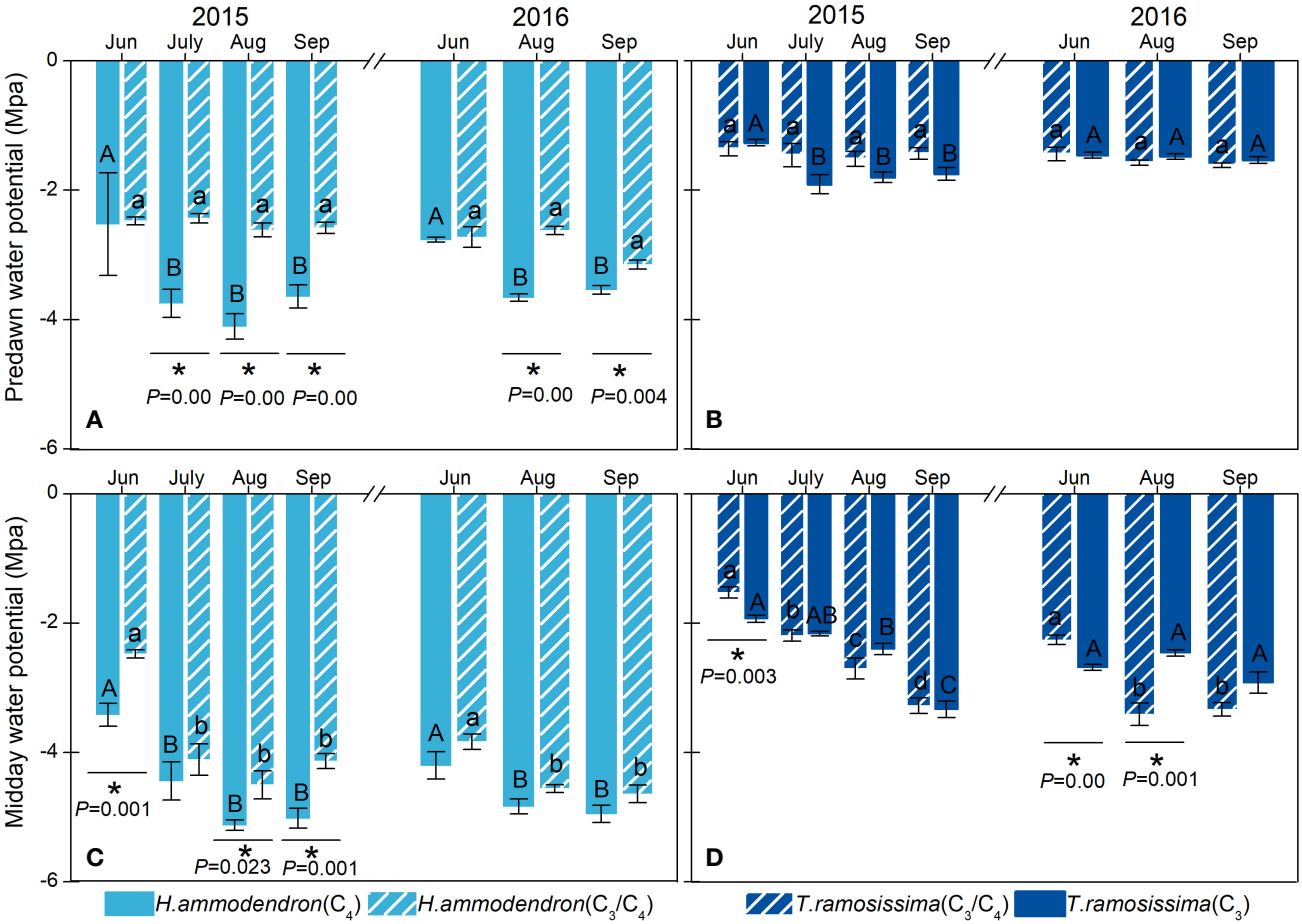
Variations in stem predawn (Ψ*_md_*) and midday water potentials (Ψ*_pd_*) of *Haloxylon ammodendron* **(A, C)** and *Tamarix ramosissima* **(B, D)** in different sites during the growing seasons of 2015 and 2016. The different uppercase and lowercase letters represent monthly significant differences for given shrubs at different sites. * represents significant intraspecific differences in different sites (*p*< 0.05). Data are presented as mean ± 1 standard error (n = 6).

The Ψ*_pd_* and Ψ*_md_* of *H. ammodendron* differed significantly at the two sites. The *H. ammodendron* at the drier site (C_4_ site) had more negative water potential than the *H. ammodendron* at the wetter site from June to September. Particularly, the Ψ*_pd_* of *H. ammodendron* at the drier site dropped significantly during the drier months (**Figure 4**). However, the water potential of *T. ramosissima* did not differ significantly between the two sites during the study period, except for in June and August of 2016 (**Figure 4**).

### 3.4 Variation in *RRR*s of eco-physiological parameters of the shrubs

**Figure 5:**
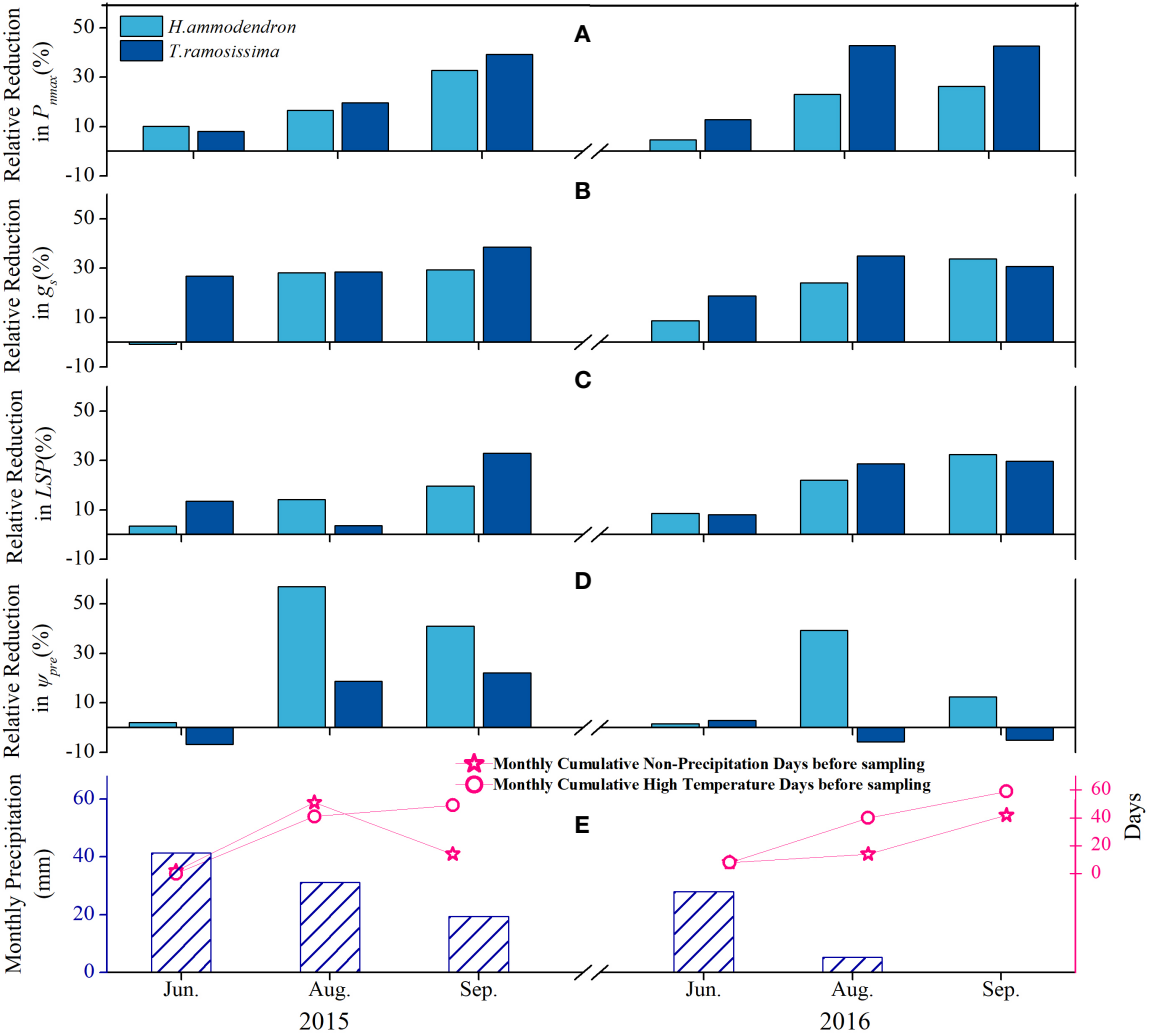
Variations of the relative reduction ratio of average *P*_*nmax*_ **(A)**, *g*_*s*_ **(B)**, *LSP* **(C)**, and Ψ_*pd*_ **(D)** of *Haloxylon ammodendron* and *Tamarix ramosissima* with the monthly cumulative non-precipitation days (MCND, panel **E**), monthly cumulative high-temperature days before sampling (MCHTD, panel **E**), and monthly total precipitation amount (MTPA, panel **E**) during growing seasons of 2015 and 2016.

The *RRR* (Equation 2) values of the *P_nmax_*, *g_s_*, and *LSP* of both shrubs increased gradually with increasing monthly cumulative non-precipitation days (MCND) and monthly cumulative high temperature days before sampling (MCHT), as well as with the decreasing monthly total precipitation amount (MTPA) (**Figure 5**). The *RRR* values of the parameters were higher for *T. ramosissima* than for *H. ammodendron*. The *RRR* of Ψ*_pd_* was higher in August and September than in June for *H. ammodendron*, whereas there was no such trend in the Ψ*_pd_* for *T. ramosissima* (**Figure 5**).

### 3.5 Responses in non-structural carbohydrates in leaves

**Figure 6:**
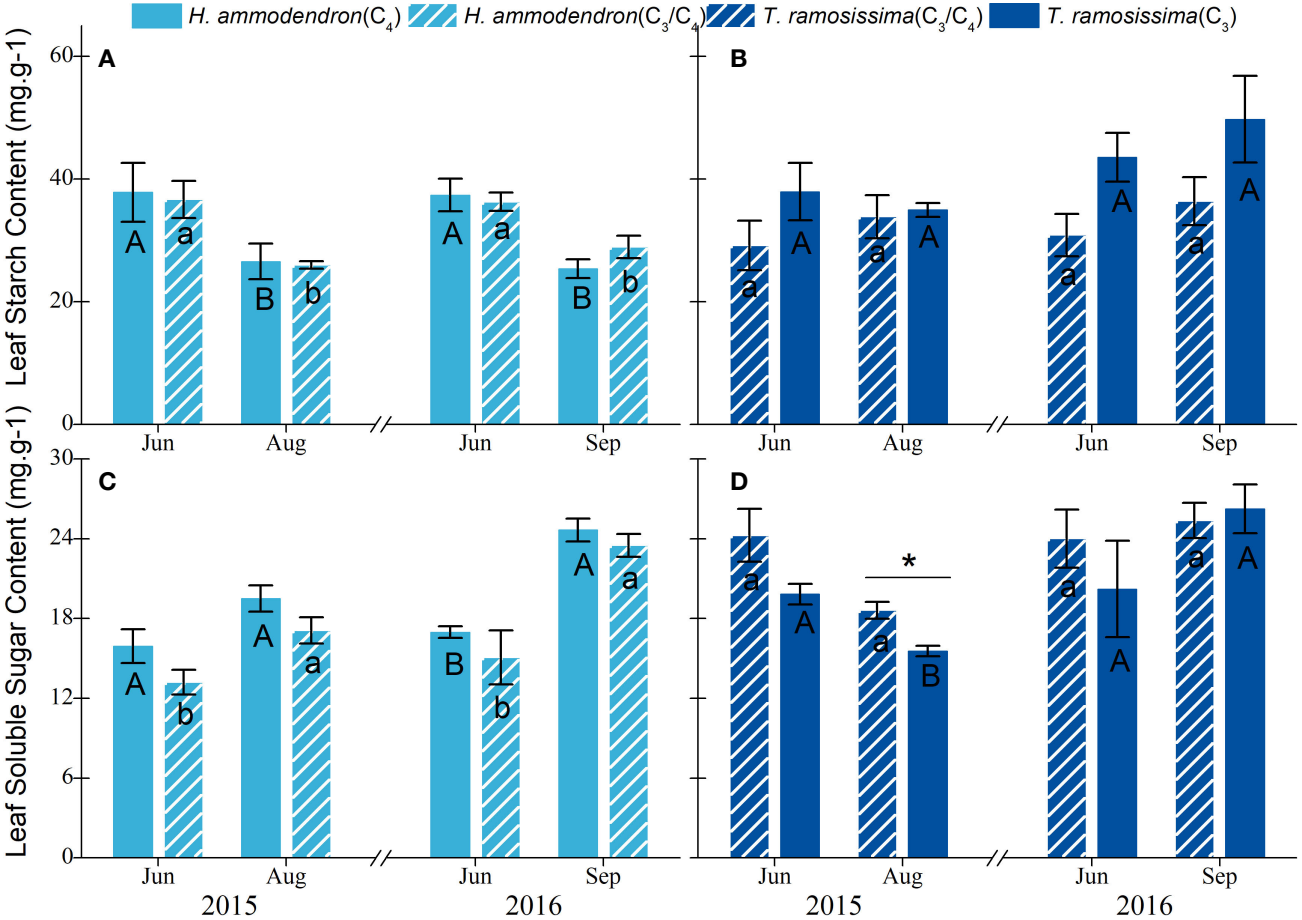
Variations in leaf starch content and leaf solute sugar content of *Haloxylon ammodendron* **(A, C)** and *Tamarix ramosissima* **(C, D)** in different communities in 2015 and 2016. The different uppercase and lowercase letters represent monthly significant differences for given shrubs at different sites. * represents significant intraspecific differences in different sites (*p*< 0.05). Data are presented as mean ± 1 standard error (n = 6).

The leaf starch content and leaf soluble sugar content of *H. ammodendron* did not vary significantly with sites (**Figure 6**). However, there were significant monthly differences observed in these two parameters at both sites. Specifically, the leaf starch content decreased significantly during drier months, whereas the leaf soluble sugar content tended to increase significantly during the drier period (*p*< 0.05, **Figure 6**). For *T. ramosissima*, the leaf starch content tended to increase during the drier months of both years and increased significantly during August 2016 (**Figure 6**). However, the leaf-soluble sugar content of *T. ramosissima* showed a decreasing trend at the drier site, but a statistically significant decrease was only detected in August 2015 (**Figure 6**). The NSCs of *T. ramosissima* did not differ by month in both sites during both years (**Figure 6**).

## 4 Discussions

This study demonstrated that the two studied Central Asian desert shrubs with differential photosynthetic pathways performed differently with declining groundwater depth.

### 4.1 Responses of *H. ammodendron* to the declining groundwater depth

The diminishing groundwater had a great impact on the water use, gas exchange, and stem water status of the Central Asian desert shrubs. The water sources of *H. ammodendron* reverted from the soil water (at the C_3_/C_4_ site) to the groundwater (at the C_4_ site), and the percentage of groundwater use increased with the month in response to the declining groundwater depth (**Figure 2**). These results were in line with the findings of earlier reports conducted on the same species in the same study area (Dai et al., 2015; Wu et al., 2019), which suggest that this shrub could adjust its rooting depth flexibly in space and time to cope with the water stress caused by fluctuating water conditions (Tiemuerbieke et al., 2018). In addition, the capacity to extract water from relatively stable groundwater via a strong root system would account for the ability of *H. ammodendron* to maintain relatively constant eco-physiological performance under changing water conditions (Drake and Franks, 2003; Xu and Li, 2006).

The responses of the eco-physiological parameters of *H. ammodendron* to the declining groundwater depth were manifested during the prolonged summer drought, indicating that the prolonged summer drought further intensified the effect of declining groundwater depth. At the start of the summer drought (during June), the response of *H. ammodendron* to the declining groundwater depth in terms of gas exchange was not pronounced. The *RRR* values of the *P_nmax_* and *g_s_* of the shrubs at the drier site were the lowest during this period (**Figure 5**). However, with the progress of the summer drought, with elevated high temperatures and an increasing number of non-precipitation days, the *RRR*s of *P_nmax_*, *g_s_*, and *LSP* increased substantially during the following months, and the highest values were observed in September (**Figure 5**). The same trends were detected in the predawn water potential of *H. ammodendron*, which tended to be more negative at the C_4_ site during August and September (**Figure 4**). The *RRR* of the predawn water potential was greater during the chronic summer drought (**Figure 5**), suggesting that the ability of *H. ammodendron* to recover daytime water status was impaired by the prolonged summer drought at the site with deeper groundwater depth (**Figures 4**, **5**). Similar results were reported in previous studies conducted on the same species (Wu et al., 2019), indicating the pronounced impacts of the summer drought on the plant responses under changing water conditions. Moreover, the ΔΨ reflects the hydraulic balance between water loss from the leaves and supply from the roots and soil, with smaller ΔΨ at the C_4_ site (**Supplementary Figure 2**), indicating that nighttime rehydration under severe drought was insufficient to bring the Ψ*_pd_* into equilibrium with the soil water potential (Taylor et al., 2014). The present study did not detect a significant reduction in the transpiration rate of *H. ammodendron* at the site with lower groundwater depth. It can be inferred that *H. ammodendron* sustains the transpiration rate and stomatal conductance essential for growth by relying on stable groundwater. This might be a water-saving strategy for this shrub, as it allows *H. ammodendron* to respond quickly to changes in the ambient vapor pressure deficit (VPD) to prevent excessive dehydration (Chen et al., 2015).

### 4.2 Responses of *T. ramosissima* to declining groundwater depth

The carbon assimilation, stomatal conductance, and water transpiration were all suppressed at the site with deeper groundwater depth, and the relative reductions in the average photosynthetic rate and stomatal conductance were greater at the site with deeper groundwater depth and accelerated with drought, indicating that the eco-physiological performance of *T. ramosissima* was greatly affected by the combined effects of declining groundwater depth and chronic summer drought. However, unlike *H. ammodendron*, the water sources of *T. ramosissima* did not shift, and this shrub mainly relied on deep soil water at both sites (**Figure 2**). The stem water status did not differ significantly with declining groundwater depth but deteriorated with the progression of the summer drought at both sites (**Figure 4**). These results suggested that the deeper soil water might be the reliable water source that enabled this shrub to maintain comparable water status with the wetter site, but the monthly declining midday water potential indicated that the water status of this shrub would be significantly affected by progressive atmospheric desiccation at both sites.

### 4.3 Comparisons

Both species exhibited a decline in eco-physiological performance due to the reduction in groundwater depth, especially when coupled with prolonged summer drought, and the response patterns differed between the two shrubs. With declining groundwater table depth, the water sources shifted, carbon fixation and stomatal conductance were suppressed, the water status of stems further deteriorated, and the transpiration became less sensitive in *H. ammodendron*. The water sources and water status responded less sensitively in *T. ramosissima*, indicating that the two shrubs prioritized water consumption and carbon gain differently when faced with changing water conditions.

The reduced transpiration rate and stomatal conductance and increased instantaneous water use efficiency of *H. ammodendron* (**Table 1**, **Supplementary Figure 1**) together with its deep root system reflected the adoption of a typical conservative water use mechanism by the C_4_ shrub. The deep roots of *H. ammodendron* at the deeper groundwater site would guarantee a reliable water source to maintain its normal eco-physiological performance. Such a strategy seemed to be beneficial for maintaining its hydraulic functions (Zhu and Cao, 2009; Chen et al., 2015) during the dry season with high temperatures and evaporative demand. The smaller stomatal aperture and higher efficiency of water use in *H. ammodendron* were consistent with the typical pattern and classical understanding of the traits of C_4_ grass species discussed in previous reports and developed models (Kocacinar and Sage, 2003; Taylor et al., 2011; Osborne and Sack, 2012; Taylor et al., 2014). This is also the case for the Central Asian C_4_ shrub based on the present study.

**Table 1:** Inter-specific differences in the eco-physiological parameters between *Haloxylon ammodendron* and *Tamarix ramosissima* at same sites during the growing seasons of 2015 and 2016.

Parameters	2015			2016		
	June	August	September	June	August	September
*P_nmax_*	*p* = 0.274 (*F*3= 1.294)	***p* = 0.019 (*F* = 7.569)**	***p* = 0.008 (*F* = 12.086)**	*p* = 0.186 (*F* = 2.352)	***p* = 0.000 (*F* = 60.819)**	*p* = 0.116 (*F* = 3.595)
*g_s_*	***p* = 0.000 (*F* = 70.64)**	***p* = 0.000 (*F* = 33.42)**	***p* = 0.000 (*F* = 107.91)**	***p* = 0.000 (*F* = 115.19)**	***p* = 0.000 (*F* = 44.96)**	***p* = 0.007 (*F* = 14.05)**
*T_r_*	***p* = 0.002 (*F* = 16.93)**	***p* = 0.000 (*F* = 73.64)**	***p* = 0.000 (*F* = 31.11)**	***p* = 0.000 (*F* = 73.55)**	***p* = 0.000 (*F* = 51.03)**	***p* = 0.001 (*F* = 36.12)**
*LSP*	*p* = 0.374 (*F* = 0.852)	*p* = 0.867 (*F* = 0.03)	*p* = 0.901 (*F* = 0.016)	*p* = 0.654 (*F* = 0.214)	*p* = 0.083 (*F* = 3.801)	*p* = 0.613 (*F* = 0.275)
Ψ*_pd_*	***p* = 0.000 (*F* = 76.99)**	***p* = 0.000 (*F* = 48.62)**	***p* = 0.000 (*F* = 83.23)**	***p* = 0.000 (*F* = 46.79)**	***p* = 0.000 (*F* = 138.45)**	***p* = 0.000 (*F* = 289.86)**
Ψ*_md_*	***p* = 0.000 (*F* = 76.81)**	***p* = 0.000 (*F* = 43.49)**	***p* = 0.000 (*F* = 25.78)**	***p* = 0.000 (*F* = 114.42)**	***p* = 0.000 (*F* = 39.51)**	***p* = 0.000 (*F* = 49.12)**

Significant differences are in bold.

As discussed above, strict stomatal regulation of the C_4_ shrub could be the key water conservation strategy to limit excessive water loss. *H. ammodendron* may obtain a great advantage by adopting this water-saving mechanism. On the one hand, the effective regulation of stomata in *H. ammodendron* is essential for sustaining hydraulic function in hot and dry conditions. Additionally, the transpiration rate in *H. ammodendron* remained constant at its lowest point across the growing season, which might aid this shrub conserve water in the prolonged dry season and decrease the likelihood of stem embolism (Meinzer and McCulloh, 2013; Chen et al., 2015). On the other hand, the consequence of this avoidance of hydraulic failure is the subsequent limitation of carbon gain that occurs with tight stomatal control (McDowell and Sevanto, 2010; Silvertown et al., 2015). Nevertheless, the CO_2_-concentrating mechanism within the C_4_ shrub seemed to compensate for the CO_2_ limitation caused by tight stomatal control (Ripley et al., 2007; Osborne and Sack, 2012). According to previous studies, rapid reduction in atmospheric CO_2_ concentrations and elevated ambient temperatures have selected the evolution of the C_4_ pathway (Taylor et al., 2010; Osborne and Sack, 2012), which eliminates the limitation of photorespiration by elevating CO_2_ concentrations at the site of the bundle sheath where Rubisco fixes CO_2_ (Ripley et al., 2010; Sage, 2014). This allows C_4_ plants to maintain the same rate of photosynthetic efficiency at a lower stomatal conductance compared with C_3_ ancestors (Taylor et al., 2012; Liu et al., 2019). As revealed in this study, the *P_nmax_* of *H. ammodendron* did not differ significantly compared with *T. ramosissima* at the same site during the rapid growth month (June) in both years (**Figure 3**, **Table 1**), demonstrating that *H. ammodendron* exhibited higher carbon fixation, with stomatal conductance being approximately half that of *T. ramosissima*. Additionally, it can be inferred from the *A*–*Ci* curves that the reduced *P_nmax_* of *H. ammodendron* might result from metabolic limitations. *H. ammodendron* seemed to be more susceptible to metabolic limitations under declining groundwater depth than *T. ramosissima* (**Supplementary Figure 3**), which aligns with the results of previous reports on C_3_ and C_4_ grasses (Ripley et al., 2007).

**Figure 7:**
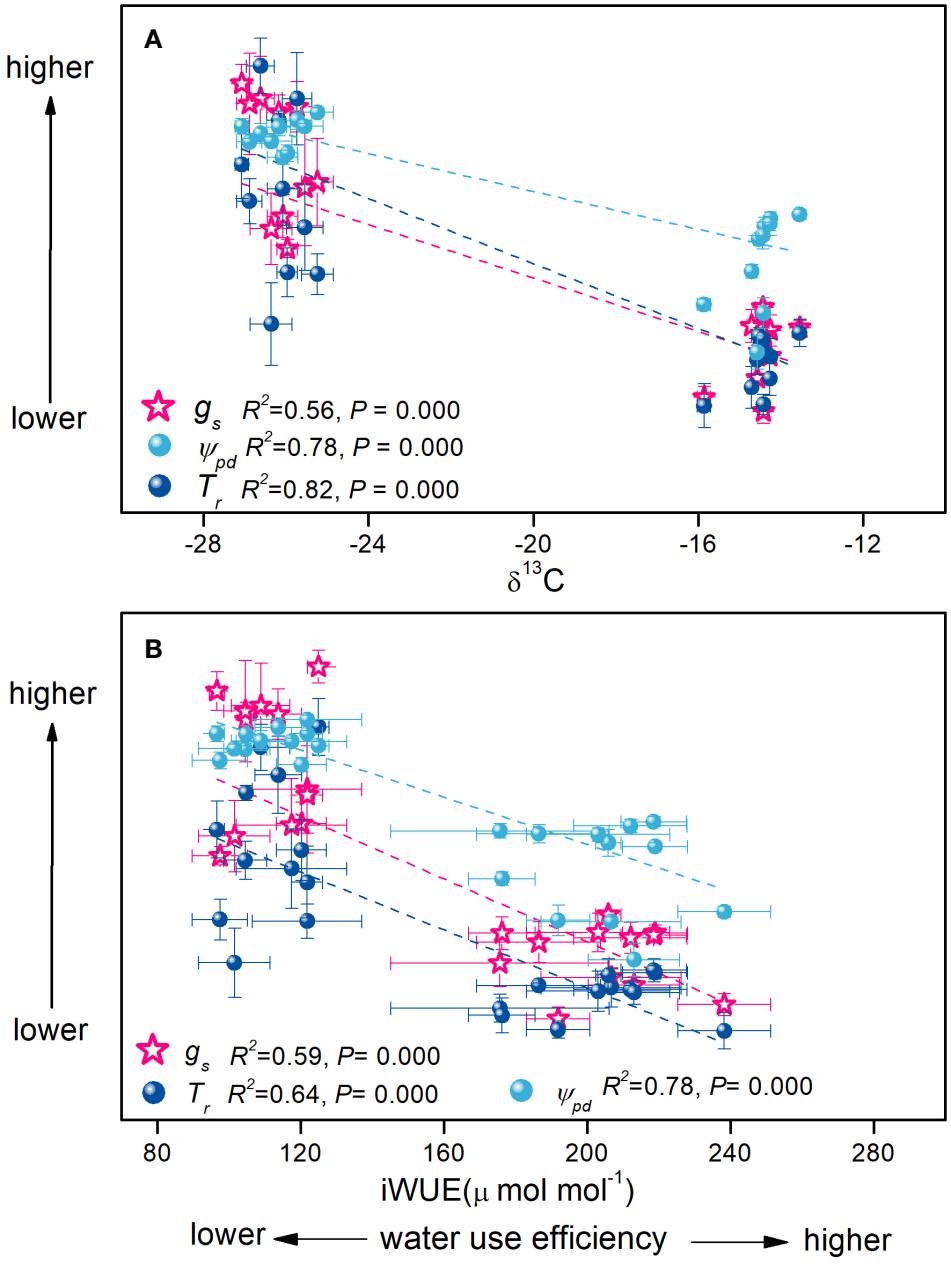
Relationships of δ^13^C **(A)** and intrinsic water use efficiency **(B)** with stomatal conductance, transpiration rate, and predawn water potential of the *Haloxylon ammodendron* and *Tamarix ramosissima* during the growing seasons of 2015 and 2016. Data were pooled for all individuals of both shrubs at two sites during the whole study period. R^2^ and *p*-values are given (*p*< 0.05).

Contrary to *H. ammodendron*, the C_3_ shrub *T. ramosissima* adopted a contrasting water–carbon balance. *T. ramosissima* maintained higher stomatal conductance during the growing season. Consequently, the transpiration rate and photosynthetic rate were higher (**Table 1**), indicating that *T. ramosissima* might guarantee a higher carbon fixation rate at the great cost of higher water loss and lower water use efficiency (**Figure 7**). This was also consistent with the earlier research on the same shrub in the same study region, which suggested that *T. ramosissima* has the tendency to assimilate carbon at its maximum level, at the risk of greater water loss (Xu et al., 2007). However, the higher *RRR* in gas exchange parameters (**Figure 5**) indicated the downregulation of its eco-physiological performance with declining groundwater depth. Retaining greater stomatal conductance under a declining groundwater table gradient might make this shrub more susceptible to hydraulic failure, which might be the main factor determining its absence in the central areas of the desert, where the groundwater table is even lower. Therefore, *H. ammodendron* achieved a higher rate of carbon fixation with greater water use efficiency (lower stomatal conductance) than the C_3_ shrub *T. ramosissima* to which a higher rate of carbon assimilation comes with far greater stomatal conductance.

Additionally, the stem water status of the two shrubs was affected differently by declining groundwater depth. The stem water status of *T. ramosissima* was more sensitive to the progressing summer drought than the declining groundwater table, whereas both the groundwater table and the prolonged summer drought strongly affected the water status of *H. ammodendron*. It could be inferred that the two shrubs might adopt different stem hydraulic and morphological properties to guarantee the water transport in stems under changing water conditions. *H. ammodendron* could tolerate greater negative water potential with the decline in groundwater table depth, which might be associated with specific stem morphology and hydraulic properties (such as different xylem morphological traits, stem hydraulic conductance, and stem vulnerability). Previous reports have pointed out that plants with lower water potentials have smaller and thicker xylem conduits with fewer pits specialized in membranes (Lens et al., 2013). These plants are able to handle low water potentials without experiencing embolism, which allows them to safely transport water to the leaves even in extremely dry environments at near-maximum rates (Manzoni et al., 2013). These embolism-resistant stems would guarantee hydraulic safety for these shrubs under unfavorable conditions.

When data were pooled for all individuals of both shrubs at two sites during the entire study period, the greater negative stem water potential of *H. ammodendron* coupling with lower transpiration rate and stomatal conductance were associated with improved instantaneous water use efficiency and more positive *δ* ^13^C values. The higher water potential of *T. ramosissima* coupled with its higher stomatal conductance and transpiration rate was associated with lower instantaneous water use efficiency and more negative *δ* ^13^C values (**Figure 7**). Therefore, it could be inferred that the evaluated carbon fixation of *T. ramosissima* was accompanied by the coordination with more favorable stem water status and higher water conductance from the leaf to the atmosphere. However, the progressive atmospheric desiccation during summer drought might greatly inhibit this process. For *H. ammodendron*, the water conservation mechanism seemed to rely on the coordination of the stomatal behavior, photosynthetic pathway, and stem hydraulic properties. Tight stomatal regulation was coordinated with an embolism-resistant stem that could tolerate greater negative water potentials. This was in line with previous reports that have proposed that plants’ regulation of stomatal behavior could be in balance with the leaf water potential above a threshold value that limits excessive water loss to avoid hydraulic failure in the xylem to maintain the water-carbon balance (Sperry, 2000; Meinzer et al., 2009; Klein, 2014; Roman et al., 2015). Furthermore, the reduction in stomatal conductance would not necessarily give rise to a marked reduction in photosynthetic assimilation due to the C_4_ pathway (**Figure 5**). Therefore, it is suggested that the evolution of stem hydraulic traits occurred in coordination with the evolution of photosynthetic pathways so that water conductance would keep pace with water consumption. As suggested by Taylor et al. (2014), the diversification in plants’ hydraulic traits may have been crucial in the colonization and adaptation of C_4_ grasses to arid and open environments. This was also the case for C_4_ and C_3_ desert shrubs in the Gurbantunggut Desert, where *Haloxylon* species were mainly distributed at the fringe of the desert, whereas the C_3_ species were distributed at the edge of the desert.

**Figure 8:**
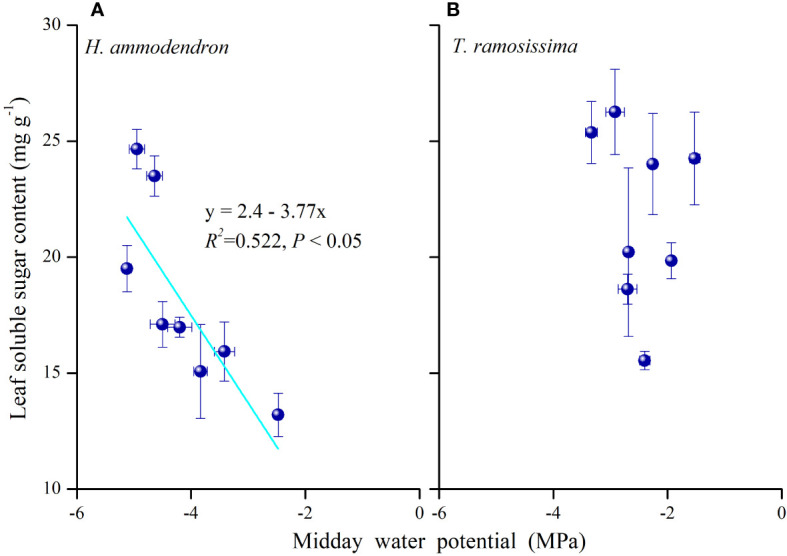
Relationship between leaf soluble sugar content and midday water potential of *Haloxylon ammodendron* **(A)** and *Tamarix ramosissima* **(B)** in 2015 and 2016. Data were pooled for the two plants. R^2^ and *p*-values are given (*p*< 0.05).

The partitioning pattern of leaf NSCs differed between the two shrubs. The significant monthly variations in the non-structural carbohydrates of the two shrubs demonstrated different carbon allocation processes in the two shrubs. The significant negative association of leaf soluble sugar content with midday water potential in *H. ammodendron* (**Figure 8**) revealed that the reduction of leaf starch content and the significant increase in leaf solute sugar content in drier months may be the result of osmotic or turgor regulation of the leaf. This suggested that when leaf water potentials gradually became negative during dry periods, more osmotically active carbohydrates such as sugars were partitioned to the leaf to maintain leaf turgor and leaf hydraulic water status in *H. ammodendron* (Woodruff et al., 2015).

## 5 Conclusions

The two studied shrubs demonstrated contrasting trade-offs between water loss and carbon gain under water stress and changing water conditions. Tight stomatal regulation was beneficial for *H. ammodendron* due to its C_4_ photosynthetic pathway, which seemed to compensate for the constraints on carbon fixation caused by lower stomatal conductance during the growing season. A relatively strong rooting system made this shrub more resilient with the declining groundwater table. On the contrary, *T. ramosissima* maximized carbon gain at the cost of lower water use efficiency by maintaining constantly greater stomatal conductance across the growing season. However, this might make *T. ramosissima* more vulnerable to hydraulic failure under the declining groundwater gradient. Marked differences in water status between the two shrubs suggested that different stomatal behaviors might coordinate with contrasting hydraulic properties in the two shrubs to maintain the balance between the water conductance and water loss, which would mitigate competitive stress by separating their hydrological niches and facilitate their coexistence.

## Data availability statement

The original contributions presented in the study are included in the article/**Supplementary Material**. Further inquiries can be directed to the corresponding author.

## Author contributions

BT: Conceptualization, Funding acquisition, Investigation, Methodology, Formal analyses, Writing – original draft, Visualization. J-YM: Conceptualization, Funding acquisition, Provision of measurements and data, Writing – review & editing. WS: Writing – review and editing. All authors contributed to the article and approved the submitted version.
