# Supplementary material for: Differential eco-physiological performance to declining groundwater depth in Central Asian C3 and C4 shrubs in the Gurbantunggut Desert
Source: Front Plant Sci. 2024 Jan 18;14:1244555. doi: 10.3389/fpls.2023.1244555 (PMC10835802; doi:10.3389/fpls.2023.1244555)
Supplement: Supplementary file 1 [file DataSheet_1.docx]

Table S1. Vegetation characteristics of two desert shrubs in three communities

| Communities | Species | Species Height (cm) | Rooting depth | Groundwater table depth | Photosynthetic type |
| --- | --- | --- | --- | --- | --- |
| C_4_ shrub community | *H. ammodendron* | 263 ± 14.9 | ≈1000 cm | 10 m | C_4_ |
| C_3_/C_4_ shrub mixed community | *H. ammodendron*  *T. ramosissima* | 265 ± 20.7  266 ± 20.7 | ≈300 cm  ≈300 cm | 3.5 m | C_4_  C_3_ |
| C_3_ shrub community | *T. ramosissima* | 277 ± 10.1 | Unknown | 6 m | C_3_ |


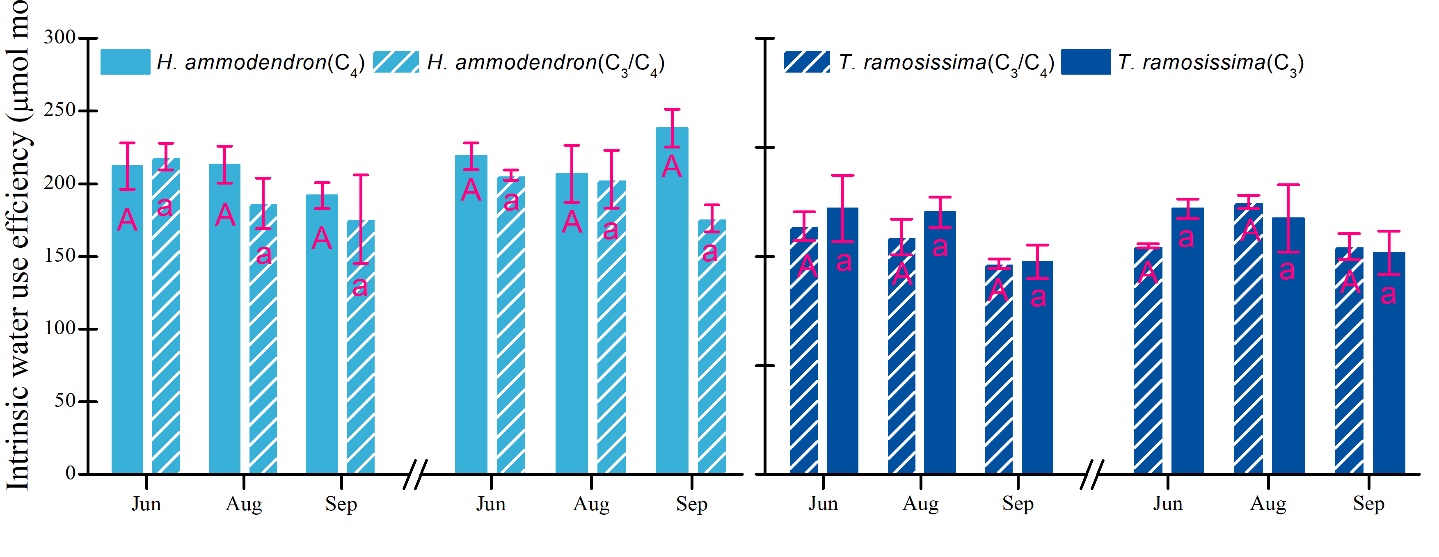
Figure S1. Variations in intrinsic water efficiency (i*WUE*) of *H. ammodendron* and *T. ramosissima* in different sites during the growing seasons of 2015 and 2016. The different uppercases and lowercases represent monthly significant differences for given shrub at different site. * represents significant intraspecific difference in different sites (*P* < 0.05). Data are presented as mean ± 1 standard error (n=6).


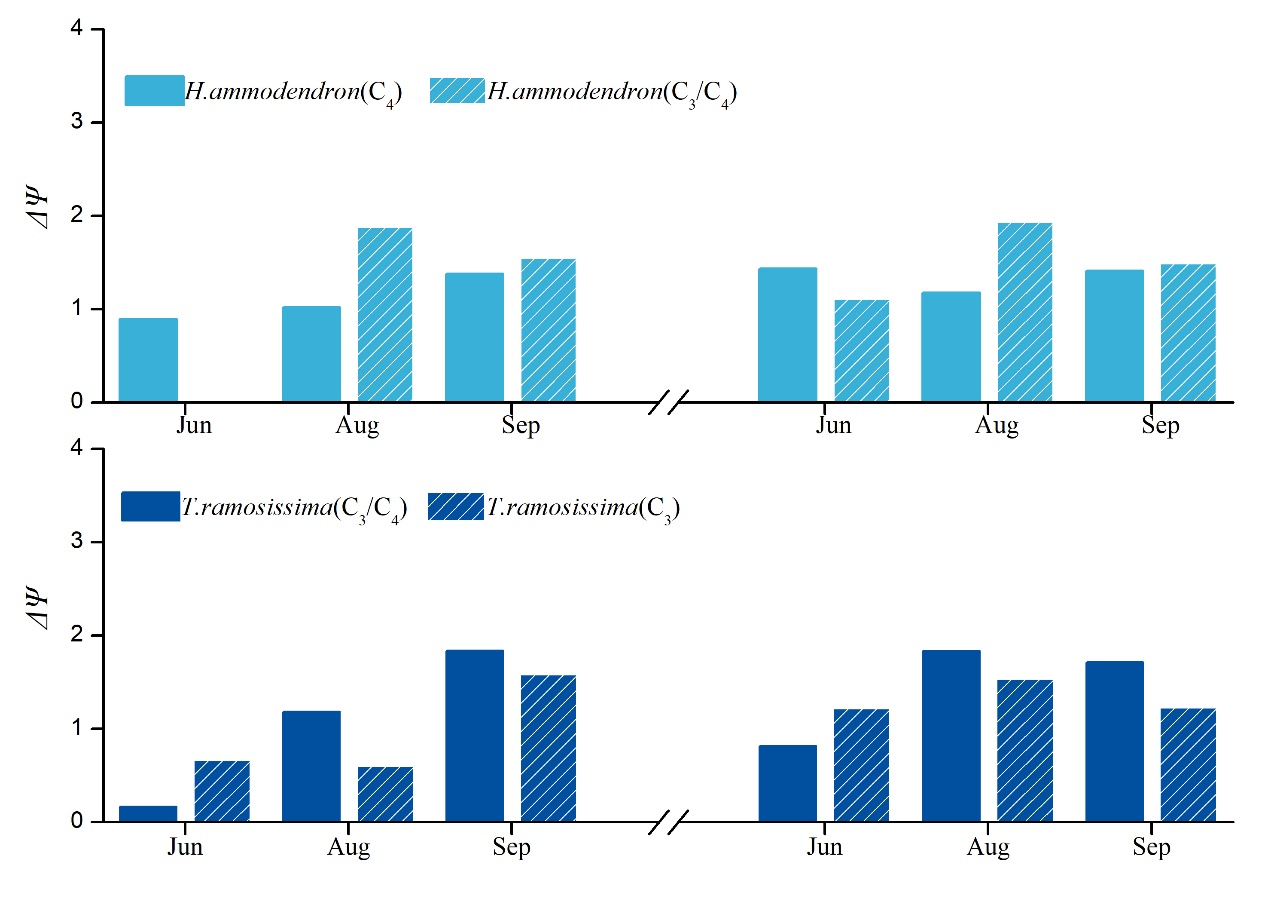
Figure S2. Differences between predawn and midday water potential (**ΔΨ**) of *H. ammodendron* and *T. ramosissima* in different sites during the growing seasons of 2015 and 2016.


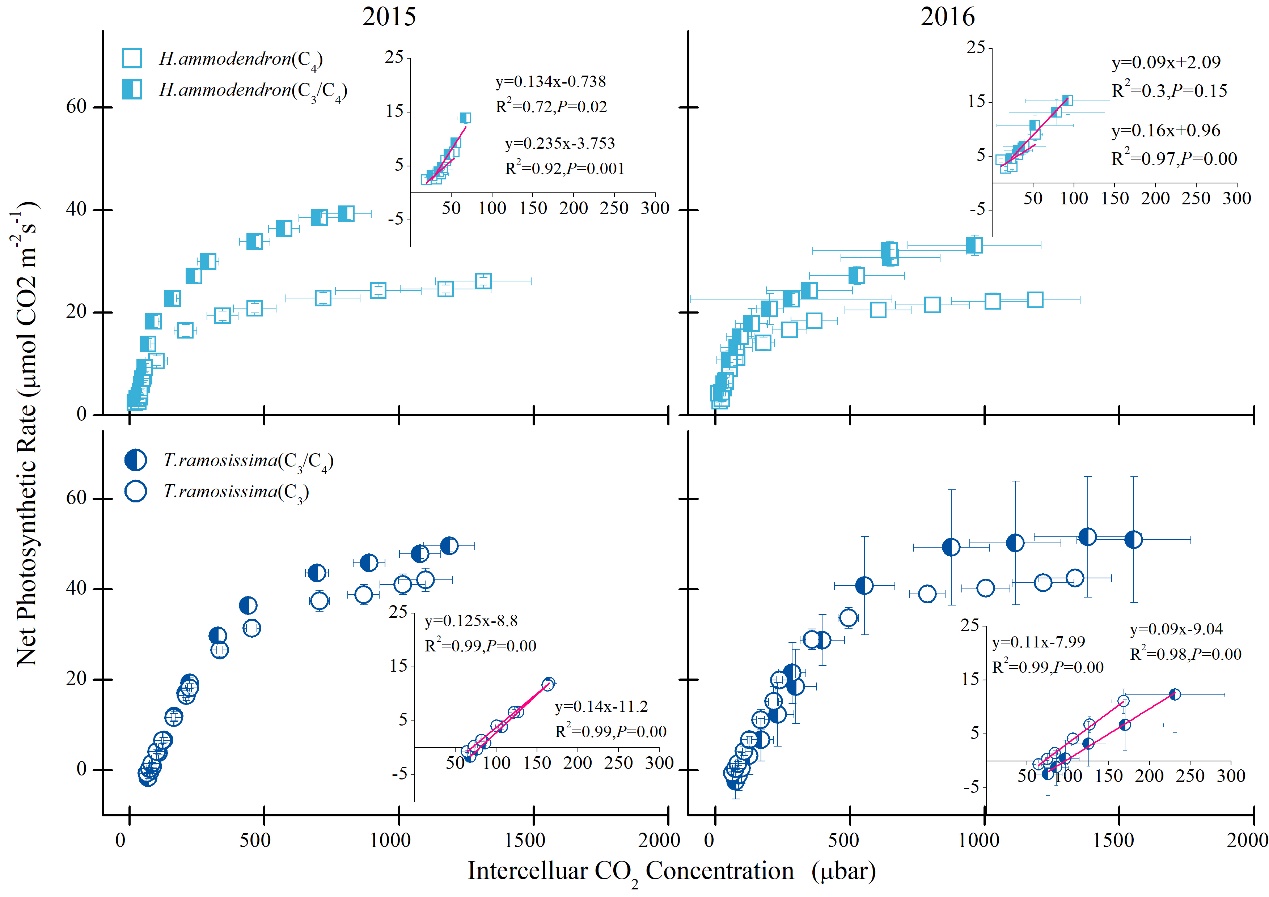
Figure S3. Responses of the net photosynthetic rate to intercellular CO_2_ concentration for the two shrubs. Inset shows the initial responses of net photosynthetic rate to chloroplast CO_2_ concentration.
